# Alzheimer’s Disease: Understanding Motor Impairments

**DOI:** 10.3390/brainsci14111054

**Published:** 2024-10-24

**Authors:** Jesús Andrade-Guerrero, Humberto Martínez-Orozco, Marcos M. Villegas-Rojas, Alberto Santiago-Balmaseda, Karen M. Delgado-Minjares, Isaac Pérez-Segura, Mauricio T. Baéz-Cortés, Miguel A. Del Toro-Colin, Magdalena Guerra-Crespo, Oscar Arias-Carrión, Sofía Diaz-Cintra, Luis O. Soto-Rojas

**Affiliations:** 1 Laboratorio de Patogénesis Molecular, Laboratorio 4, Edificio A4, Carrera Médico Cirujano, Facultad de Estudios Superiores Iztacala, Universidad Nacional Autónoma de México, Tlalnepantla 54090, Mexico; jesusandrade1007@gmail.com (J.A.-G.); marcusvillegas3@gmail.com (M.M.V.-R.); albertosantiago@comunidad.unam.mx (A.S.-B.); km95.delgado@gmail.com (K.M.D.-M.); dr.isaacps91@gmail.com (I.P.-S.); mau.ldz.floyd@gmail.com (M.T.B.-C.); angelcolinn007@gmail.com (M.A.D.T.-C.); 2 Departamento de Neurobiología del Desarrollo y Neurofisiología, Instituto de Neurobiología, Universidad Nacional Autónoma de México, Querétaro 76230, Mexico; h_martinez@live.com.mx; 3 Sección de Estudios de Posgrado e Investigación, Escuela Superior de Medicina, Instituto Politécnico Nacional, Ciudad de México 11340, Mexico; 4 Departamento de Fisiología, Biofísica y Neurociencias, Centro de Investigación y de Estudios Avanzados del Instituto Politécnico Nacional, Ciudad de México 07360, Mexico; 5 Laboratorio de Medicina Regenerativa, Departamento de Fisiología, Facultad de Medicina, Universidad Nacional Autónoma de México, Ciudad de México 04510, Mexico; mguerra@facmed.unam.mx; 6 Unidad de Trastornos del Movimiento y Sueño, Hospital General Dr. Manuel Gea González, Ciudad de México 14080, Mexico; ariasemc2@gmail.com

**Keywords:** Alzheimer’s disease, motor impairments, neuromuscular, muscle atrophy, strength loss, gait, balance, coordination, posture, cardiorespiratory fitness

## Abstract

Alzheimer’s disease (AD), the most prevalent neurodegenerative disorder and the leading cause of dementia worldwide, profoundly impacts health and quality of life. While cognitive impairments—such as memory loss, attention deficits, and disorientation—predominate in AD, motor symptoms, though common, remain underexplored. These motor symptoms, including gait disturbances, reduced cardiorespiratory fitness, muscle weakness, sarcopenia, and impaired balance, are often associated with advanced stages of AD and contribute to increased mortality. Emerging evidence, however, suggests that motor symptoms may be present in earlier stages and can serve as predictive markers for AD in older adults. Despite a limited understanding of the underlying mechanisms driving these motor symptoms, several key pathways have been identified, offering avenues for further investigation. This review provides an in-depth analysis of motor symptoms in AD, discussing its progression, potential mechanisms, and therapeutic strategies. Addressing motor symptoms alongside cognitive decline may enhance patient functionality, improve quality of life, and support more comprehensive disease management strategies.

## 1. Introduction

Alzheimer’s disease (AD) is the most common cause of dementia and the leading neurodegenerative disorder globally, affecting approximately 50 million individuals. The World Health Organization (WHO) projects that by 2050, the number of AD cases will triple, establishing AD as a global public health priority. In 2019, dementia-related care incurred a global cost of 1.3 trillion dollars, with approximately 50% of these expenses shouldered by informal caregivers, who play a critical role in managing the high dependencies of patients. These dependencies, which stem from cognitive and physical impairments, intensify as the disease progresses into its intermediate and late stages (WHO 2023) [1].

Researchers have identified two types of AD: the familial form, which manifests early and accounts for only 1–5% of cases, and the sporadic form, which occurs in individuals over 65 and accounts for about 95% of cases [2]. The term “AD continuum” is commonly used to describe the gradual and progressive development of the disease, from its earliest signs to its advanced stages. The AD continuum classifies the disease according to the progression of symptoms into three stages: cognitively normal or presymptomatic, mild cognitive impairment, and dementia. The histopathological Braak and Braak stages and the Thal phases correlate with these disease stages [3,4].

Two distinctive neuropathological markers characterize AD: neurofibrillary tangles (NFTs), which are made of hyperphosphorylated tau protein, and extracellular aggregates of amyloid-beta (Aβ) peptide, which accumulate in brain tissue to form neuritic plaques and in blood vessels to form cerebral amyloid angiopathy (CAA) [5]. These markers primarily affect areas related to memory, such as the hippocampus; however, they are not limited to these regions, as their spatiotemporal progression impacts other anatomical areas related to motor control, such as the motor cortex, cerebellum, and basal ganglia, causing motor impairments in patients and, consequently, a higher degree of disability [6].

While cognitive deficits, such as impaired cognitive function, memory problems, disorientation, and learning difficulties, are the most well-known and commonly studied signs and symptoms of AD, numerous studies indicate that motor system impairments are also typical [7]. Motor deficits in AD become particularly pronounced during the intermediate and late stages, though current evidence suggests they can also emerge in the early stages [8]. Although researchers have not studied these changes as extensively as cognitive issues, their importance is evident due to their direct impacts on patient functionality and quality of life. These motor deficits are even linked to significant mortality events, including pneumonia and infection processes [9,10].

Among AD’s most common motor deficits are gait disturbances, commonly known as “cautious gait” [8]. AD patients have an increased risk and incidence of falls, which can cause serious injuries [11]. Researchers have observed problems with coordination and manual dexterity, along with deficits in dynamic and static balance, which may be related to the previously described issues [8]. Other motor signs, such as bradykinesia, rigidity, and motor denervation, can also be present in AD patients [12]. Furthermore, some of the most frequent motor signs are muscle atrophy and decreased strength, which have been found even in the early stages of the disease and progress with it [13]. Studies have correlated the strength decrease with reduced brain volume and cognition, as well as with abnormal weight loss and cachexia, which directly impact patient functionality and increase the risk of fractures [14,15].

Motor impairments contribute to movement restriction and the limitation of physical activities imposed by caregivers or family members for the patient’s well-being, resulting in patients being less active than they are when in a physiological state [16]. This process, in turn, reduces their cardiorespiratory capacity, leading to lower maximum oxygen volume (VO_2_max), which is associated with an increased risk of mortality and poor cognitive performance [17,18].

Motor deficits in AD remain poorly understood, with limited insights into the mechanisms driving these impairments [6]. The relationship between the progression of neuropathological features and the emergence of motor dysfunction is still unclear [19]. As these deficits are often under-recognized or deprioritized in research, this review aims to address this gap by exploring the onset, the anatomopathological and physiological progressions, and the potential predictive biomarkers of motor impairments in AD. We highlight the need for the comprehensive integration of motor assessments from clinical studies and animal models while identifying therapeutic avenues and future research opportunities to advance this critical aspect of AD pathology.

## 2. Spatiotemporal Progression of AD Symptoms and Their Connection to Motor Impairments

Motor signs in AD usually result from pathological changes in the extrapyramidal system; however, their exact anatomical location is not precise [20]. Clinically, some recognized molecular alterations in AD, like those in tau or Aβ, are involved in anatomical changes in different brain regions and are valuable markers to determine the AD stage and diagnosis (Table 1). For instance, in the presymptomatic stage of the disease, the transentorhinal region of the temporal mesocortex (Braak and Braak stage I) and the CA1 and CA2 subregions of the hippocampus (Braak and Braak stage II) are the first brain regions to exhibit conditions related to alterations caused by tau proteins [21,22]. Similarly, in the Thal phases, the brain regions mainly affected by Aβ are the neocortex (Thal phase 1), entorhinal region, CA1, insular cortex, amygdala, cingulate gyrus, and the presubicular region (Thal phase 2) [23]. These alterations cause subtle changes in thinking abilities that are first noticed by the individual when cognitive tests do not reveal significant evidence of objective impairment [24]; they are apparent as alterations in episodic memory and verbal memory recall [25]. Although researchers have long considered that neuropathological changes begin in the cortex, they have observed pre-tangle accumulation in subcortical regions [3], such as the locus coeruleus (LC) and nucleus basalis of Meynert (NBM) [26]. This process can explain the neuropsychiatric symptoms observed in the disease before the development of cognitive impairment, such as depression, anxiety, and sleep disturbances [26,27].

Interestingly, researchers have observed that motor alterations, including motoric cognitive risk syndrome, tremor, and restless leg syndrome, precede cognitive decline [28]. Damage to the cholinergic projection pathways from the pedunculopontine nucleus and NBM explains these alterations, with the former innervating the basal ganglia and thalamus to control gait and posture directly [3,29,30]. The amygdala directly connects with the motor cortex, subthalamic nucleus, and globus pallidus, making it crucial for controlling motor function [29,31]. Researchers have associated these connections with the appearance of aberrant motor behavior [32,33].

During the prodromal stage of the disease, tau pathology progresses to the temporal association areas, entorhinal cortex, and parahippocampal, occipitotemporal, and lingual gyri (Braak and Braak stage III), as well as to the hippocampus CA3 and CA4, insular cortex, thalamus, claustrum, and medial temporal gyrus (Braak and Braak stage IV) [21,22]. Meanwhile, in Thal phase 3, the Aβ pathology extends to subcortical regions and the midbrain, such as the caudate nucleus, putamen, claustrum, basal forebrain nuclei, substantia innominata, diencephalon, superior and inferior colliculus, CA4, and red nucleus [23]. In this stage, memory impairment becomes evident; the decrease in episodic memory and verbal memory recall [34] manifests through difficulty acquiring new tasks, fluent aphasia, and apraxia [35]. The alterations in other structures of the neocortex and the insular cortex are related to the appearance of neuropsychiatric symptoms: agitation, anxiety, appetite dysfunction, irritability, euphoria, and disinhibition [27,32]. In this stage, the most remarkable motor symptoms in AD patients are decreased limb strength and changes in postural control [28,35]. These symptoms correlate with the alterations in structures that participate in motor function control, such as the substantia nigra, basal ganglia, and thalamus [36,37,38], which leads to the presence of falls and the most significant deterioration in the ability to perform instrumental activities of daily living [35].

Finally, in the dementia stage, tau pathology is observed in larger areas of the cortex, such as the superior temporal gyrus, premotor area, and primary temporal association areas (Braak and Braak stage V), followed by the prostriata and striatum association areas of the occipital neocortex (Braak and Braak stage VI) [21]. In contrast, Aβ deposition is present in different brainstem nuclei, such as the inferior olivary nucleus, substantia nigra, and reticular formation of the medulla oblongata (Thal phase 4); moreover, it occurs in the reticular formation and reticular tegmental nucleus of the pons, Raphe nuclei, LC, parabrachial nuclei, and dorsal tegmental nucleus (Thal phase 5) [23]. Cognitive symptoms, executive and visuospatial dysfunction, and alterations in language characterize this stage [39]. Additionally, neuropsychiatric symptoms include apathy, delusions, and hallucinations [27,40].

Regarding motor symptoms, this stage presents slow essential mobility, impaired balance and gait, and difficulties in performing dual tasks [40,41]. Researchers have observed alterations in areas related to the initiation of appendicular and facial movements, including the medial frontal gyrus, precentral gyrus, gyrus rectus, and anterior cingulate cortex. These changes are associated with the progression of Parkinsonism and the development of truncal and facial dyskinesias [42,43,44]. In addition, tau [45,46] and Aβ pathology have also been observed in neurons of the anterior medullary horn and the cerebellum, causing dysmetria, ataxia, muscle weakness, and spasticity [23,47]. All of the above hinder the patient from performing the basic activities of daily living due to the need for adequate motor and cognitive functions to perform them [35].

Given the increasing evidence linking motor impairments to AD, it is essential to prioritize their study alongside cognitive decline, as motor symptoms significantly affect the quality of life and functionality of patients. Future research should focus on understanding the anatomical and pathophysiological mechanisms underlying these impairments, particularly in the early stages. Integrating motor assessments in clinical studies and animal models can provide valuable insights into AD progression. Moreover, early identification of predictive biomarkers for motor symptoms can also enhance diagnosis and treatment, making comprehensive care for cognitive and motor symptoms vital for improving the lives of those affected by AD.

**Table 1:** Association between Alzheimer’s disease continuum, Braak and Braak stages, and Thal phases.

Alzheimer’s Disease Continuum			
	**Cognitively Healthy or Presymptomatic**	**Mild Cognitive Impairment**	**Dementia**
**Cognitive** **symptoms**	↑ Subjective cognitive impairment (alteration in episodic and verbal memory)	↑ Memory impairment (decrease in episodic and verbal memory)	↑ Executive and visuospatial dysfunction ↑ Alterations in language
**Neuropsychiatric symptoms**	↑ Depression, anxiety, and sleep disturbance	↑ Agitation, anxiety, appetite dysfunction, irritability, euphoria, and disinhibition	↑ Apathy, delusions, and hallucinations
**Motor symptoms**	↑ Motoric cognitive risk syndrome, tremors, gait disturbances, restless leg syndrome, and aberrant motor behavior	↑ Parkinsonian symptoms (rigidity, bradykinesia and postural instability), ↓ Strength, muscle mass, CRF levels, and changes in postural control	↑ Aberrant and slow motor behavior, balance, and gait problems, speech-facial expression dual-task difficulties, and Parkinsonian symptoms
**Braak and Braak stages**			
	**I–II**	**III–IV**	**V–VI**
Brain regions affected	NCx, EC, CA1, IC, AMG, CG, MFG, PreS	EC, PHG, OTG, LG, HC-CA3, HC-CA4, IC, Thal, Cla, MTG	STG, HAA, ProA, StrA
**Thal phases**			
	**1–2**	**3**	**4–5**
Brain regions affected	NC, HC-CA1, EC	CN, Put, Cla, BFN, SI, Thal, Hyp, LHN, CS, CI, CA4, RN, STN	ION, SN, RFMO, RFP, ARN, CRN, LC, PBN, DTN, RTNP, Cb, RtTg

Abbreviations: AMG: amygdala, ARN: anterior raphe nuclei, BFN: basal forebrain nuclei, CA1: Ammon’s horn CA1, CRF: cardiorespiratory fitness, CI: colliculus inferior, Cla: claustrum, CG: cingulate gyrus, Cb: cerebellum, CN: caudate nucleus, CRN: caudal raphe nuclei, CS: colliculus superior, DTN: dorsal tegmental nucleus, EC: entorhinal cortex, HAA: primary temporal association areas, HC-CA3: hippocampus CA3, HC-CA4: hippocampus CA4, Hyp: hypothalamus, IC: insular cortex, ION: inferior olivary nucleus, LC: locus coeruleus, LG: lingual gyrus, LHN: lateral hypothalamic nucleus, MFG: medial frontal gyrus, MTG: medial temporal gyrus, NCx: neocortex, PBN: parabrachial nuclei, PHG: parahippocampal gyrus, PreS: presubicular region, ProA: prostriata association areas, Put: putamen, RFMO: reticular formation of the medulla oblongata, RtTg: reticulo tegmental nucleus of the pons, RN: red nucleus, SI: substantia innominata, SN: substantia nigra, STG: superior temporal gyrus, STN: subthalamic nucleus, StrA: striata association areas, ↑: increase, and ↓: decrease. The information in the table was obtained from [21,22,26,27,28,29,45].

## 3. Motor Impairments in Alzheimer’s Disease

### 3.1. Gait Disorders

Gait is a complex task that, although often considered automatic, requires continuous adjustments to maintain control of the body’s position during movement [48]. This process relies on the proper integration and functioning of both sensorimotor and cognitive systems, involving motor processes and memory, cognition, attention, decision-making, and problem-solving. As a result, gait assessment plays an essential role in various physical examinations, including those for AD [48].

Gait disorders are common in aging, but in individuals with AD, deviations from normal walking are more pronounced compared to healthy peers of the same age. This exacerbation is linked to factors such as decreased muscle mass and strength, reduced muscle blood flow, limited mobility, inflammation, and oxidative stress, all of which arise from the impaired sensorimotor systems associated with AD [49,50]. Older adults with gait disorders are estimated to have a 1.2–2.5 times higher risk of developing AD [49]. In AD patients, the typical gait pattern, known as “cautious gait”, is characterized by a slower walking speed, a shorter step length, increased step variability, a wider support base, a longer double support time, and greater postural instability. These gait abnormalities emerge in the early stages of the disease and worsen as AD progresses [8,51]. Similar alterations and their pathological progressions have been observed in both AD patients and animal models, where early-stage changes deteriorate with age [52,53,54].

Researchers have correlated gait variations with cognitive batteries, such as the Montreal Cognitive Assessment (MoCA), which helps distinguish between healthy individuals and those with mild cognitive impairment (MCI) or AD [55]. Furthermore, dual-tasking gait assessment, such as conversing or counting backward while walking, offers a more accurate measure of the relationship between cognition and gait. This approach provides deeper insights into how cognitive impairment impacts gait and affects daily activities [51,56]. Variables such as step time, gait speed, sway time, double support, single support, and step length can help differentiate healthy subjects from those with MCI or AD [55].

Different reports have extensively studied gait speed and demonstrated that it predicts dementia development in individuals initially without dementia [50,57]. Reduced gait speed appears even in the early stages of the disease and directly correlates with impairments in executive functions, working memory, and an increased risk of falls [48]. Furthermore, individuals with the apolipoprotein ɛ4 (APOE4) allele experience a more pronounced decline in gait speed than non-carriers [58]. Moreover, this parameter has been associated with increased mortality in older adults, leading some authors to suggest it may be the sole parameter capable of predicting dementia, in addition to being an important biomarker in AD [51]. Therefore, evaluating gait from the early stages of the disease is crucial for identifying potential impairments that might affect functionality and possible predictive biomarkers of AD.

### 3.2. Decline in Cardiorespiratory Fitness

Cardiorespiratory fitness (CRF) is closely tied to an individual’s level of physical activity and refers to the VO_2_ max in response to the body’s energy demands [59]. Higher CRF levels are associated with better brain functions in healthy older adults. Benefits include maintaining brain and hippocampal volume, preserving white matter microstructure, reducing the incidence of cardiovascular disease, and lowering mortality rates [60,61]. Additionally, improved physical performance is associated with a reduced risk of developing dementia [62,63].

In AD patients, physical activity levels decrease due to various motor and mental impairments, as well as imposed physical restrictions for safety. Consequently, CRF levels drop, with an estimated 20% reduction compared to healthy individuals of the same age [64]. Such a decrease in CRF among AD patients correlates with several findings, including reduced brain volumes in regions such as the hippocampus, amygdala, supramarginal gyrus, and rostral middle frontal gyrus. Early-stage AD also shows decreased white matter integrity in the fronto-occipital fasciculus [65,66]. Furthermore, lower CRF levels are associated with poorer cognitive performance, executive functions, learning, memory, and visuospatial abilities and higher mortality rates [64]. CRF is also linked to Aβ42 levels, and it impacts immediate and verbal memory learning [67], with lower CRF levels found to be APOE4 allele carriers [68].

Nevertheless, maintaining higher CRF through physical activity can help mitigate cognitive impairments in AD patients, highlighting the importance of incorporating physical exercise into their care to improve overall health and quality of life. Therefore, sustaining physical activity and assessing CRF as both a predictor of AD and an indicator of overall health is crucial for managing AD effectively.

### 3.3. Muscle Atrophy and Strength Loss

Sarcopenia is a syndrome characterized by a pathological decline in muscle mass and function, which directly impacts an individual’s functionality [69]. Muscle mass and strength decline with age, reducing by 1–2% annually starting from the third decade of life and accelerating to 1.5–3% per year after age 50 [70]. Sarcopenia has a multifactorial etiology associated with chronic inflammation, insulin resistance, hormonal imbalances, malnutrition, and physical inactivity, among other factors [71]. These factors contribute to falls, fractures, disability, and even mortality [72]. Studies show that older adults with dementia and AD exhibit higher rates of sarcopenia and declining muscle strength [8,73,74]. Patients with dementia have a sarcopenia rate that is 3–5 times higher than that in adults without dementia [75].

Additionally, healthy adults with higher rates of sarcopenia have a 1.58 times greater likelihood of cognitive impairment [71]. Research using dual-energy X-ray absorptiometry (DEXA) has shown that muscle mass loss related to sarcopenia is evident from the early stages of the disease [19,73]. This loss is correlated with cognitive decline, reduced brain volume in areas such as the frontal lobe, amygdala, and hippocampus, and decreased cerebral blood flow [76,77]. Furthermore, studies have found that the presence of sarcopenia increases the likelihood of developing AD by 197% and any other type of dementia by 58% [71]. There is also a preferential loss of type II muscle fibers related to muscle power [75].

On the other hand, AD patients show a decline measured by tests such as the handgrip test. This decline is evident from the early stages of the disease, becomes more pronounced in the intermediate stages, and worsens as the disease progresses compared to control groups [13,72]. This parameter is also associated with cognitive impairment, particularly in memory and attention, and it can be a risk predictor for cognitive decline. It is linked to previously described gait disturbances, affecting the patient’s functionality and quality of life [78,79]. Monitoring handgrip strength is thus necessary as a marker of functionality and cognition in older adults and patients with AD [74]. Animal model studies reflect a decrease in muscle mass from the early stages of the disease, progressing with the development of pathology. These studies also show deficiencies in muscle strength, which are evident in the intermediate and late stages of the disease [19,53]. Therefore, the positive association between sarcopenia, decreased strength, and neurocognitive disorders in AD is clear. Nonetheless, both parameters should be evaluated and included in the disease assessment.

### 3.4. Disruptions in Balance

Balance refers to the body’s ability to remain upright and stable while performing movements (dynamic) or in a specific posture (static) [80]. Many studies report that in AD, balance control deteriorates as cognitive impairment increases [81]. Patients with an AD diagnosis have a 44% higher risk of falls compared to control patients, along with impairments in various static and dynamic balance conditions. Activity level, gait, and mobility are also affected, particularly during turning and balance maintenance tasks [82].

A study evaluated balance control in patients with AD, MCI, and moderate AD (MAD) using the Balance Evaluation Systems Test (BESTest). The results show that the mild AD group performs worse than the MCI patient group, while the MAD patient group has the worst scores among the groups. This suggests that impairments in coordination and balance are associated with disease progression and the development of Parkinsonian symptoms commonly observed in AD patients [83].

The Timed Up and Go (TUG) test assesses balance impairment by measuring the time it takes for a patient to rise from a chair without armrests, walk three meters, and sit back down. Researchers have observed that patients with moderate MAD take longer and have slower speeds on the TUG test compared to healthy control patients, indicating balance issues [84].

Another study using the same test on patients with subjective cognitive impairment (SCI), MCI, and AD shows an increase in task completion time related to disease progression. Using the one-leg standing test (OLST), they have reported coordination impairments, showing deterioration from SCI patients, and it is more acute in patients carrying the APOE4 allele [81]. Finally, studies have associated the worst balance scores with patients who have more significant cognitive impairment based on the TUG scale, the balance subscale of the Performance-Oriented Mobility Assessment (POMA-B), and the Functional Gait Assessment (FGA) [85]. Since balance deficits impact various motor tasks and patient functionality and correlate with the severity of cognitive deficits, medical professionals should evaluate these parameters in AD patients to assess disease progression and severity.

### 3.5. Impact on Postural Impairments

Posture is the position adopted by an individual involving neuromusculoskeletal systems. As AD progresses, many motor symptoms become noticeable, including difficulties in movement planning and postural stability. A study reported that postural stability performance decreases by about 32% in older adults with cognitive impairment compared to healthy patients, according to tests with open and closed eyes [86]. Meanwhile, another study reported that AD patients exhibit greater postural instability and display distinct kinetic profiles compared to healthy controls [87]. The complex process of maintaining balance, which involves the coordination of multiple body systems, is notably disrupted in AD patients, especially under conditions of visual suppression, increasing their risk of falls. These findings highlight the crucial role of cognitive factors in postural control.

Regarding structural postural deficits, studies using animal models have found that alterations such as hyperkyphosis and clasping in the advanced stages of the disease can contribute to increased disability [52,53]. The results suggest that kinetic analysis may be a valuable tool for identifying Alzheimer’s patients at higher risk of falls [87]. However, more studies are needed to analyze postural alterations in the different stages of the disease and the possible underlying pathways of these alterations. Table 2 summarizes different findings regarding motor impairments in AD in clinical studies and animal models.

## 4. Potential Mechanisms Underlying Motor Impairments in Alzheimer’s Disease

### 4.1. Pathological Proteins and Motor Neural Pathways

#### 4.1.1. Amyloid-β Pathology

Neurodegeneration induced by Aβ pathology may play a role in exacerbating neuromuscular and motor conditions associated with aging. In this regard, AD patients with positive cerebral Aβ show motor deficits related to memory decline, suggesting a contribution of certain neurodegenerative processes, such as the atrophy of cortical brain areas [105]. However, motor deficits in disorders such as AD might precede neurodegeneration, as suggested by findings in humans [106], but the exact mechanisms remain unclear. Evidence shows that transgenic mouse models of AD, which express mutant Aβ in the human brain, also exhibit abnormal accumulation of mutant Aβ in the spinal cord or skeletal muscles [19,107,108,109,110]. Distinct alterations accompany these histopathological findings, including a decrease in the number of cholinergic neurons, demyelination, a reduction in the number of sciatic nerve fibers, denervation of neuromuscular junctions (NMJ), increased lipid peroxidation, reduced mitochondrial activity, decreased oxygen consumption rates in muscle fibers, increased transforming growth factor beta (TGF-β) signaling, sarcopenia, and decreased contractile response [19,108,109].

Moreover, in *5xFAD* mice, a progressive decline in motor behavior with age and correlated Aβ accumulation in the spinal cord are evident, but not with intracellular Aβ and neuronal loss in cortical layer V [110]. On the other hand, human studies have found Aβ accumulation in the spinal cord, although only in about 50% of cases [111]. However, researchers poorly understand the pathological role of Aβ accumulation in tissues such as the spinal cord and skeletal muscle in AD, though it may contribute to these manifestations.

Interestingly, Aβ pE 3-42, a post-translational Aβ modification, may have more cytotoxic properties. A study using the TBA2 transgenic mouse model demonstrates the immunoreactivity of this Aβ fragment in several regions, particularly in Purkinje cells in the cerebellum, which are crucial for motor functions. This region also shows significant neurodegeneration. The mice rapidly develop a clinical phenotype with the loss of motor coordination, ataxia, and premature death [112].

Therefore, the excessive production of amyloid peptides in the brain may extend to peripheral tissues and impact components of motor control, having significant implications for the progression of motor deficits in AD.

#### 4.1.2. Tauopathy

Tau pathology also contributes to the onset and progression of motor deficits in AD, as observed in transgenic mouse models of the disease, such as the *JNPL3* and *PS19* strains [113,114,115]. *JNPL3* mice carry a mutation in the microtubule-associated protein tau (*MAPT*) gene, which leads to early motor deficits that worsen with age, primarily due to spinal cord alterations, such as tau NFTs, motor neuron loss, and astrogliosis [113]. *PS19* mice carry a *P301S* mutation in the human 1N4R tau and exhibit motor deficits as early as three months of age, progressing to paralysis between seven and ten months [114]. In particular, motor dysfunction in *PS19* mice is associated with widespread tauopathy in the motor neurons of the spinal cord, resulting in their loss, axonal degeneration, and astrogliosis and inducing structural changes in muscles and myofibrils that lead to motor neuron denervation [114].

However, some authors have noted that evidence from these tauopathy mouse models should be taken with caution, as studies in humans have not correlated the presence of tau with motor deficits [116,117,118], and events such as spinal cord pathology and motor neuron loss have been under-studied in AD patients [119]. This may suggest that tauopathies might play a minor role in motor deficits in most cases of AD. However, further studies are needed to explore the potential mechanisms by which tau can influence motor impairments in the disease.

#### 4.1.3. Mixed Proteinopathy

Mixed proteinopathy in AD refers to the coexistence of multiple types of misfolded proteins in an individual, adding to the disease’s complexity and heterogeneity and possibly influencing the pathogenesis of the motor symptoms that develop in AD patients. The main proteinopathies implicated in this condition are TDP-43 protein inclusions, the accumulation of Aβ plaques and NFTs composed of hyperphosphorylated tau protein, and α-synuclein (α-syn)-associated pathology [120].

α-Syn is both a soluble presynaptic protein and the main component of Lewy bodies found in the brains of patients with Parkinson’s disease, a leading motor disorder [121]. A postmortem study found that 51.8% of AD patients show α-syn inclusions, with 34% in the substantia nigra pars compacta (SNpc) and 28% in the LC. Some of these patients exhibit motor symptoms, suggesting a possible link between α-syn in these motor regions and the motor symptoms in AD [122]. Another study involving 82 sporadic AD patients, diagnosed according to the Consortium to Establish a Registry for Alzheimer’s Disease (CERAD) criteria, found α-syn-positive structures in 32% of these patients, with equal involvement of the substantia nigra and amygdala complex [123]. In the transgenic *hSyn/hAPP* mouse model, researchers observed α-syn inclusions in the temporal and cingulate cortices without directly assessing motor areas. They also detected significant degeneration of cholinergic neurons in the caudoputamen nucleus, accompanied by motor deficits starting at 6 months of age. This suggests that *hSyn/hAPP* accelerates α-syn-dependent motor deficits in the presence of Aβ [124]. Although studies have not yet explored the impact of α-syn on muscle in AD models, synucleinopathy models suggest that α-syn has physiological and pathological functions. Despite the apparent involvement of α-syn, its pathogenesis, progression, and clinical impact in motor manifestations of AD remain unclear, emphasizing the need for further studies to clarify these aspects [125].

TDP-43 protein, typically known for its accumulation in cytoplasmic inclusions in amyotrophic lateral sclerosis, appears in phosphorylated and truncated forms—critical features of the disease [126]. Recently, studies have identified TDP-43 inclusions in aging and cognitive decline, particularly in AD, where these inclusions are present in up to 57% of cases [127,128]. Models of TDP-43 progression in advanced stages reveal pathology in motor regions, including the ventral striatum, basal ganglia, SNpc, and frontal cortex. These findings suggest a potential link between TDP-43 pathology and motor impairment in AD patients [127].

In the *5xFAD* transgenic AD mouse model, researchers found TDP-43 accumulating in the inner mitochondrial membrane of cortical layer 5, though the specific cortical region remains unidentified. This accumulation is linked to weight and muscle mass loss and gait and balance impairments, suggesting that mitochondrial TDP-43 contributes to motor dysfunction. Inhibiting this accumulation may help reverse these deficits [129]. Another study showed that TDP-43 injection in *APP/PS1ΔE9* mice significantly increases amyloid plaque load in the olfactory bulb, amygdala, and several cortical areas, including the prefrontal, motor, and somatosensory cortices [130].

To date, human studies have not found a correlation between TDP-43 progression and motor function in AD, representing an important area of opportunity. The above evidence suggests that these mixed proteinopathies present in motor regions may interact synergistically, contributing to motor dysfunction in AD [120]. These interactions remain poorly understood in terms of their roles in neurodegeneration and motor dysfunction. Further research is needed to clarify how these factors influence the motor alterations observed in some AD patients.

### 4.2. Neuronal Degeneration and Synaptic Loss Impact on Motor Function

Neuronal degeneration in AD not only contributes to cognitive decline but may also play a role in the onset of motor symptoms [131]. A human study found a significant correlation between gait dysfunction in patients with advanced AD and atrophy in specific brain areas, such as the motor cortex, middle cingulate gyrus, anterior insula, and anterior lobe of the cerebellum, using volumetric and diffusion tensor imaging (DTI) through magnetic resonance imaging (MRI) [132].

Cerebellar atrophy is another characteristic of sporadic AD, initially affecting parts of the cerebellar regions connected to the default mode network. As atrophy extends to the cerebellum’s anterior lobe, patients may exhibit motor dysfunctions, such as gait deficiencies and limb coordination issues [133,134].

On the other hand, synaptic dysfunction is an early and critical feature of AD associated with cognitive impairment [135]. This dysfunction involves synapse loss, dendritic abnormalities, and enlarged presynaptic terminals [136]. The progressive loss of synapses may affect motor areas, leading to motor dysfunction in the later stages of AD [136]. Another meta-analysis examined the effects of AD on various synaptic markers in crucial regions involved in motor control, including the motor cortex, cerebellum, and basal ganglia [137].

Researchers observed decreased presynaptic markers in the motor cortex, including synaptophysin and synaptobrevin. The cerebellum showed a more pronounced reduction in cytoskeletal proteins, while postsynaptic markers vary in their decline. The basal ganglia also reduce postsynaptic markers [137]. These findings underscore the significance of synaptic dysfunction in these regions and its potential link to motor alterations in AD.

### 4.3. Vascular Changes and Cerebral Blood Flow Impact on Motor Function

AD causes significant neurovascular unit dysfunctions, including abnormal vasoconstriction in arterioles, which reduces tissue oxygenation, and inappropriate vasodilation, which diverts blood away from regions with high metabolic demand [138]. These changes have been linked to damage in several motor areas, even in the early stages of the disease, leading to previously described motor alterations, notably gait disturbances such as reduced speed, poor dual-task performance, and apraxia [139]. Moreover, white matter pathologies that are typically caused by alterations in vascular tone, including myelin pallor, reduced axonal density, blood–brain barrier breakdown, spongiosis, and dilated perivascular spaces, are frequently observed in regions traversed by the corticospinal tract. These changes are consistently associated with motor alterations and likely contribute to the gait disturbances or generalized slowing observed in elderly AD patients [29,138,140].

CAA, characterized by the deposition of Aβ in the walls of cortical blood vessels, is associated with widespread ischemic injury, including white matter lesions and microinfarcts [141], in regions such as the supramarginal gyrus, superior frontal gyrus, and inferior temporal gyrus. These regions are essential for cognitive and motor functions [142]. Researchers have linked CAA, neurovascular unit disturbances, and structural alterations in blood vessels, such as perivascular edema, to impaired motor performance in an animal model [143]. Vascular changes have also been identified in regions like the cerebellum and striatum—areas particularly susceptible to small vessel disease and high blood pressure, especially in meningeal vessels and occasionally in the brainstem. These alterations may contribute to balance and coordination disturbances [138]. These findings highlight the critical role of preserving vascular and white matter integrity to maintain motor function in AD [144,145,146].

### 4.4. Motor Pathway Alterations Implicated in Motor Dysfunction

Numerous pyramidal and extrapyramidal motor deficits emerge during the course of AD, accompanying cognitive decline [29]. Researchers have widely reported the deposition of Aβ and NFTs in several cortical areas, including the primary motor cortex, supplementary motor areas, and the white matter of the spinal cord, particularly in the corticospinal tract [36,147]. In animal models, studies found damage to corticospinal axons, showing progressive dilation as the disease developed. They also observed that Aβ deposition in the spinal cord co-localized with this axonal damage and associated projection areas in *TgCRND8* mice [97,148]. Another study observed the onset of amyloid plaques in both gray and white matter of the spinal cord in the *5xFAD* mouse model, with plaques primarily deposited in the spinal white matter, particularly in the ventral part of the dorsal column corresponding to the corticospinal tract in rodents [103].

Human studies reported neuropathological findings in early-onset familial AD due to an *N135S/PSEN1* mutation. These patients present cognitive and motor deficits, such as spastic dysarthria, limb spasticity, and seizures. Autopsy findings include evidence of corticospinal tract degeneration [147]. Another study identified tau immunoreactivity in neurons of the anterior horn of the spinal cord in AD patients, noting a lesser extent in the intermediate zone and posterior horn [45,46,149].

Using DTI, researchers identified increased geometric microstructural properties of white matter fiber orientation around the lateral ventricles, particularly in the corpus callosum and parts of the corticospinal tract in AD. This increase may result from neuronal loss, glial swelling, and the subsequent impact on enlarged perivascular spaces [150].

Current findings indicate a complex interaction between the corticospinal tract and motor dysfunction in AD, but further research is needed to fully understand its impact, underlying mechanisms, and the rate of progression. Additionally, investigating other motor pathways contributing to motor deficits is crucial, as the corticospinal tract is likely not the only affected system. Identifying these additional tracts and understanding their roles may offer a more comprehensive view of the motor deficits associated with AD and inform more targeted therapeutic approaches.

### 4.5. Cholinergic Dysfunction

Acetylcholine (ACh) is a crucial neurotransmitter for cognitive processes in the brain, but it also controls motor functions and modulates neuromuscular activity [151]. A decrease in acetylcholinesterase (AChE) activity has been reported in free mitochondrial fractions of skeletal muscle in *3xTg*-AD mice at 3, 6, and 12 months old compared to age-matched controls [104]. Additionally, the authors found that AChE activity in skeletal muscle is similar between non-transgenic 12-month-old mice and 6-month-old *3xTg*-AD mice, indicating that AD pathology promotes the early decrease in muscle AChE activity that manifests with aging. A recent report has provided the first evidence of cholinergic denervation in the skeletal muscle of 6-month-old *Tg2576* mice, accompanied by a reduction in the expression of nicotinic ACh receptors (nAChRs) and choline acetyltransferase [95]. Although none of these studies evaluate motor functions, it is evident that AD promotes early impairments in ACh metabolism and signaling in skeletal muscle, which are crucial for neuromuscular transmission.

Changes in the vesicular ACh transporter (vAChT), which is expressed in nerve terminals and modulates the transport of this neurotransmitter, are essential for motor deficits in AD. For example, a recent positron emission tomography study in healthy subjects aged 20 to 80 who were administered an [18F]-labeled vAChT ligand found a relationship between older age and reduced ligand binding to vAChT in several brain regions involved in motor function control [152]. Previously, reductions in vAChT binding affinity with other ligands, such as 5-aminobenzovesamicol, have also been found in the temporal cortex of AD patients and elderly healthy subjects [153]. Furthermore, this study’s binding affinity values in AD patients are positively correlated with choline acetyltransferase activity in this brain region, suggesting deficits in ACh synthesis and transport. Previous research in mice with reduced vAChT levels has yielded interesting data [154]. The authors demonstrated decreased expression levels of these transporters in the cortex, striatum, spinal cord, and hippocampus, leading to impaired neuromuscular transmission. This finding suggests that vAChT deficiency disrupts ACh transport from the brain to the muscles. These findings point to motor dysfunction in AD involving the disruption of cholinergic transmission from the brain to the NMJ due to defects in ACh transport.

### 4.6. Peripheral Nerve and Neuromuscular Dysfunction

Aging affects communication between neurons and muscles primarily due to structural changes in the NMJ, including denervation, NMJ instability, increased axonal degeneration, and motor neuron death. These changes are partly due to the alteration in the denervation–reinnervation cycles of skeletal muscles, impacting the components of NMJs [155]. Animal studies have found axonopathy and abnormalities in the microstructures of the myelin sheaths, including progressive and significant swelling at the peripheral level, which suggests the presence of myelinopathy in the disease [97,103]. Myostatin, also known as growth differentiation factor 8, is a cytokine of the skeletal muscle and a member of the TGF-β superfamily that acts as a negative modulator of myogenesis, affecting muscle growth and size [156,157]. Myostatin deficiency regulates skeletal muscle metabolism, mitochondrial function, motor axonal growth, motor unit size, and muscle innervation; this protein’s expression may significantly contribute to motor deficits in AD [158,159]. In APP/PS1 mice, increased myostatin expression in the gastrocnemius muscle produces atrophy, while elimination through short hairpin RNA treatment promotes muscle mass and grip strength [99].

**Figure 1:**
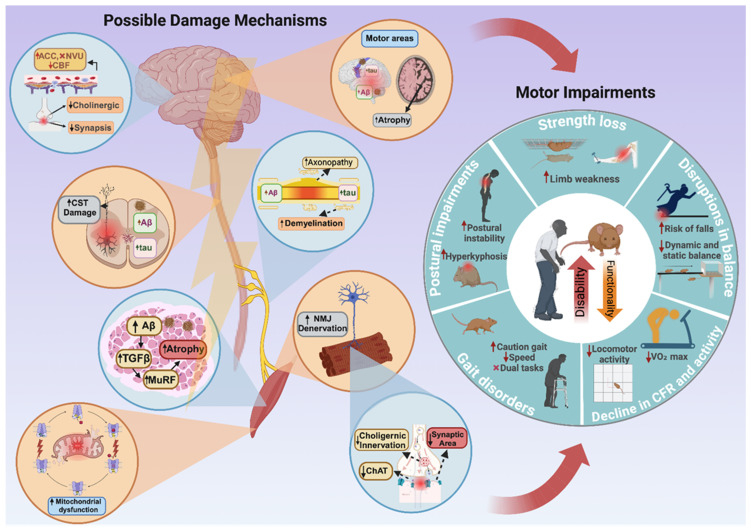
Potential brain damage mechanisms and related motor symptoms in Alzheimer’s disease. Abbreviations: Aβ: Amyloid beta; ACC: Cerebral amyloid angiopathy; CBF: Cerebral blood flow; CRF: Cardiorespiratory fitness; ChaT: Choline acetyltransferase; CST: Corticospinal tract; MuRF: Muscle RING-finger protein; NMJ: Neuromuscular junction; NVU: Neurovascular unit; ROS: Reactive oxygen species; TGFβ: Transforming growth factor beta; VO₂max: Maximum oxygen volume; ↑: Increase; and ↓: Decrease. This figure was created with BioRender.com (accessed on 9 October 2024).

Interestingly, a recent clinical study found an association between higher serum levels of myostatin and lower Aβ ratios in the brains of older adults, suggesting that myostatin may become a potential biomarker for AD risk [160]. Unfortunately, these studies did not evaluate locomotor activity despite myostatin’s critical role in the neuromuscular system. Future research should incorporate physical evaluations when studying myostatin and other disease markers. According to a previous report, myostatin and its precursors also colocalize with Aβ in skeletal muscle because they can form complexes with this peptide [161]. Moreover, in vitro evidence shows that the precursor protein of myostatin misfolds and spontaneously aggregates as amyloid-like fibrils, inducing cytotoxicity in myoblasts [162]. The biological relevance of this event in AD remains unclear. However, it might be significant, especially since myostatin is also expressed in neurons, axons, and oligodendrocytes within the brain [163]. In this context, myostatin pathways represent an important research niche that warrants further investigation to clarify their role in motor deficits in AD patients. Figure 1 summarizes the potential mechanisms of motor damage across various movement-related structures and their possible relationship with previously described motor impairments.

## 5. Strategies and Potential Treatments for Motor Impairments in Alzheimer’s Disease

### 5.1. Drug Therapies

Pharmacological treatment for AD varies according to disease progression, with the primary goal of improving cognitive symptoms. The main drug classes used are AChE inhibitors, such as donepezil, galantamine, and rivastigmine, and N-methyl-D-aspartate (NMDA) receptor antagonists, such as memantine [164]. AChE inhibitors improve memory by increasing ACh availability. Clinicians prescribe donepezil and rivastigmine for mild to severe stages, while galantamine is recommended for mild to moderate cases. Memantine, by contrast, reduces excitotoxic neuronal damage and is used for moderate to severe cases [165]. Despite these treatments, their effects on non-cognitive symptoms, including motor alterations, remain poorly studied.

#### 5.1.1. AChE Inhibitors

In patients with early AD, treatment with donepezil restores mitochondrial respiratory function in skeletal muscle compared to untreated subjects [166]. Given that mitochondrial dysfunction is highly associated with muscle atrophy [167] and plays a role in AD development [168], donepezil may prevent muscle atrophy and neuromuscular alterations modulating mitochondrial activity. Also, it improves the number of steps, stride length, and cadence after treatment in early AD patients [169] and reduces dual-task walking costs and walking speed in elderly MCI patients [170].

On the other hand, transdermal rivastigmine improved gait velocity under dual-task conditions in patients with mild to moderate AD [171]. However, it does not significantly improve single-task gait parameters like stride length and cadence [171]. These findings suggest that rivastigmine may enhance motor function in situations requiring cognitive and motor coordination, such as walking while performing a secondary task like counting or naming animals.

Galantamine has shown potential for treating motor deficits. In vAChT knockdown mice with severe motor impairments in grip strength tasks, pretreatment with AChE inhibitors, including galantamine and physostigmine, improved performance [154]. These findings suggest that galantamine’s role as an allosteric modulator potentiating nAChR may contribute to its positive effects on motor function [172]. Like rivastigmine, galantamine improves motor performance in dual-task conditions that combine cognitive and motor tasks [173].

A new drug therapy called RJx-01, which combines galantamine with metformin, has improved muscle integrity and function markers. It also prevents NMJ denervation, helping to maintain muscle mass and strength and ultimately enhancing physical performance in mouse models of sarcopenia caused by accelerated aging [174].

These observations suggest that drugs capable of inhibiting AChE activity, particularly in skeletal muscle, and restoring ACh levels and transport in the terminal nerves of NMJs can be potentially effective against motor deficits in AD. However, there is no evidence related to such mechanisms in mouse models of AD treated with this type of drug, and more research is needed to fully understand their impact and optimize their use for improving motor function in patients.

#### 5.1.2. NMDA Receptor Antagonist

Glutamate is the primary excitatory neurotransmitter in the brain, and its dysregulation leads to excitotoxicity, which is highly associated with memory impairment and neuronal loss in AD [175]. Glutamate transporters are also expressed in the NMJ, suggesting that glutamatergic signaling in skeletal muscle plays a role in motor functions. However, the underlying mechanisms remain unclear [176]. Remarkably, no evidence is focused on glutamatergic signaling in the NMJ for AD. At the central level, glutamate excitotoxicity in AD is prevented by treatment with memantine, contributing to alleviating cognitive impairment [177]. Interestingly, one study showed in the *Tg4-42* transgenic AD mouse model that chronic memantine partially benefits motor performance, reducing latency to fall in the balance beam task [178]. More research is needed to determine the potential of drugs targeting glutamatergic signaling on motor deficits in AD patients and preclinical models.

Finally, due to the presence of neuropsychiatric alterations, antipsychotic and antiepileptic medications may be prescribed [179]. However, motor deficits associated with these treatments have been observed. For instance, valproic acid, carbamazepine, clonazepam, and phenytoin are associated with movement disorders that can be alleviated by dose reduction or discontinuation of these medications [180]. Similarly, antipsychotics induce movement disorders in patients with AD, known as extrapyramidal side effects, some of which, such as bradykinesia, may worsen with the coadministration of acetylcholinesterase inhibitors [181]. Therefore, a better understanding of motor deficits or movement disorders in AD is crucial for improving pharmacological interventions and reducing side effects and the exacerbation of motor disturbances.

### 5.2. Non-Pharmacological Therapy for Motor Impairments in AD

#### 5.2.1. Physical Exercise

Numerous studies have proposed physical exercise as a non-pharmacological therapeutic measure that positively impacts the development of AD pathophysiology and is associated with a reduced risk of developing it [16,182,183,184]. Exercise benefits both cognitive and non-cognitive symptoms of AD. It reduces neuropathological markers, promotes angiogenesis, increases cerebral blood flow, and enhances neurogenesis, synaptogenesis, and the production of neurotrophic factors, leading to cognitive improvements. These changes translate into better functionality, psychological well-being, physical performance, and overall quality of life for patients [16,185,186,187].

Additionally, exercise has shown positive effects on the motor aspects of AD. Studies in patients demonstrate that exercise improves balance, gait, and strength, reduces the number of falls, and increases muscle mass and bone mineral density [188,189,190]. Studies in animal models have shown that exercise interventions improve sensorimotor activity and increase muscle mass and nuclei, suggesting that exercise can restore various motor impairments of AD [191]. Although more research is needed to clarify the mechanisms of exercise on motor alterations, the significant role of exercise in preventing and treating the disease is evident, highlighting the need to meet recommended levels of physical activity [186,192].

#### 5.2.2. Alternative Treatments

There is a growing trend toward using alternative therapies like acupuncture to treat health issues, including AD [193]. Studies have shown that acupuncture alleviates several hallmarks of AD, such as neuroinflammation, oxidative stress, cholinergic signaling, tauopathy, and apoptosis in the brain [193]. These effects may contribute to cognitive improvements, with some evidence suggesting that acupuncture may be more effective and safer than conventional medications [194]. Neuroimaging studies also highlight acupuncture’s efficacy in motor-associated networks [195]. However, only a few studies have specifically explored acupuncture’s contribution to motor function in AD patients. Based on these findings, acupuncture may hold promise for addressing motor deficits in AD, but further research is needed.

On the other hand, the effects of other non-pharmacological therapies, such as photobiomodulation, transcranial magnetic stimulation, and transcranial direct current stimulation, on the disease’s motor symptoms are unknown. In patients with Parkinson’s disease, non-invasive brain stimulation may be associated with increased reserve in the motor domain, helping to maintain motor functionality despite the progression of the disease, representing an essential area for research and therapeutic application [196,197].

#### 5.2.3. Dietary and Nutraceutical Interventions

Chronic dietary interventions (~14 months), such as caloric restriction or intermittent fasting in *3xTg*-AD mice, have also delayed AD pathology and increased locomotor activity [198]. Additionally, dietary interventions can have a direct impact on preventing sarcopenia in the disease [199], highlighting the benefits of nutritional interventions as a complementary therapy for AD-related motor dysfunction. A study in *3xTg*-AD mice demonstrated that chronic treatment from the third to the twelfth month of age with the epinutraceutical bioproduct nosustrophine produces several neuroprotective effects on AD pathology and improved motor coordination [200]. Therefore, early interventions at different stages of the disease with nutraceuticals and protein-enriched diets represent another potential therapeutic strategy against motor symptoms by delaying disease progression and counteracting muscle mass loss.

## 6. Perspectives and Conclusions

This review examines motor symptoms in AD, including deficits in gait, cardiorespiratory fitness, strength, coordination, balance, and posture. Although these impairments are more pronounced in advanced stages, clinicians can detect them early in AD. Early identification and monitoring of motor symptoms in AD patients are crucial, as they may reflect significant disruptions in both central and peripheral motor control pathways. Recognizing motor biomarkers and incorporating motor assessments into AD management could enhance diagnostic accuracy, improve patient outcomes, and reduce healthcare costs, although further research in this underexplored area is needed.

Accurate in vivo diagnosis of AD remains a challenge, prompting the development of various imaging techniques, plasma and neurochemical biomarkers, and cognitive batteries for detection across disease stages. However, incorporating gait, CRF, strength, and muscle mass assessments can provide an essential predictive tool for AD development. These assessments offer several advantages, including cost-effectiveness, ease of application, and low financial burden. As such, motor signs should be implemented as biomarkers and integrated into clinical practice for staging AD. More importantly, they can predict functionality and disability, identifying critical windows for prevention and treatment.

Addressing motor symptoms in AD remains a therapeutic challenge. Future treatments or novel drug developments must target motor control pathways and include motor behavior assessments to maximize efficacy. Focusing on motor outcomes can significantly improve patients’ functionality and quality of life and reduce mortality. Targeting motor impairments will help decrease disability and lower disease management costs. Primary prevention initiatives are essential, as healthy lifestyle habits and physical exercise play a critical role in mitigating risk factors for AD.

## Figures and Tables

**Table 2:** Motor impairments in Alzheimer’s disease: Findings from patients and animal models.

Characteristics of the Subjects	Studied Parameters	Main Findings	Author /Year
**Clinical Studies**			
NC, AD, LBD, and VD, both genders (65–85 years) *n* = 1789 (Japan)	-Balance -Posture	↑ Postural alterations in all types of dementia. -AD patients exhibit postural alterations in dynamic and static balance tasks.	[88] 2024
Older adults with dementia *n* = 3774 (Korea)	-Balance	-Correlation of balance problems with the risk of developing AD or VD.	[89] 2024
NC, MCI, and AD, both genders (74.2 ± 5.7 years) *n* = 121 (USA)	-Correlation of brain volumes and motor function	-Correlation of volumetric and cognitive predictors of motor learning.	[90] 2023
NC, MCI, and AD, both genders (55–84 years) *n* = 100 (Belgium)	-Vestibular function -Balance	↑ Vestibular deficits in groups with increasing cognitive impairment. ↑ Alterations in dynamic and static balance are related to cognitive impairment.	[85] 2023
MCI and AD, both genders (76.1 years) *n* = 26 (USA)	-Vestibular function -Balance -Hippocampal volume	↑ Vestibular alterations in both groups. ↓ Independence in instrumental activities of daily living. ↓ The volume of the left hippocampus and its correlation with vestibular alterations.	[91] 2022
Patients at risk of AD APOE ε4 carrier, both genders (40–65 years) *n* = 155 (USA)	-CRF	↑ Association of CRF with a slower decline in the cognitive domains of verbal learning, visual learning memory, and spatial memory. -Stronger effects among men than women.	[18] 2020
Mild AD, Moderate AD, both genders *n* = 339 (Europe)	-Gait -Risk of falls	↓ Relation of gait speed to cognitive deficit only in the early stages of the disease. -Decreased gait speed associated with an increase in falls.	[50] 2020
NC, MCI, and AD, both genders *n* = 295 (Korea)	-Balance	↑ Balance associated with the cognitive state. ↑ Balance alterations in APOE ɛ4 carriers.	[81] 2020
NC, Early AD, Mild AD, Moderate AD, both genders (*n* = 90) (USA)	-Muscle mass -Gait -Strength	↓ Gait speed correlated with the stage of the disease. ↑ Sarcopenia from early stages. -Correlation of sarcopenia index with cognitive status.	[8] 2018
Patients with AD, LBD, and VD, both genders *n* = 55 (Italy)	-Motor functionality -NP signs -Cholinergic dysfunction	↑ Locomotor impairment and extrapyramidal signs. Correlation of motor alterations with Aβ42 but not with t-tau and p-tau. ↑ Degeneration of the cholinergic system mediated by amyloid pathology.	[30] 2018
Patients with early AD in both genders (*n* = 40) (USA)	-CRF -Brain volume	↓ CRF and its correlation with lower white matter integrity in the fronto-occipital fasciculus.	[92] 2016
NC, Moderate AD both genders (72.9 ± 4.7 years) *n* = 26 (USA)	-Balance -Gait	↑ Alterations in static and dynamic balance. ↓ Gait speed and step length. ↑ Gait support time.	[84] 2015
NC, early AD, both genders (+60 years) *n* = 90 (USA)	-CRF -Brain volume	↓ CRF and correlation with progression of dementia severity in AD. ↑ Brain and hippocampal atrophy in patients with low CRF levels.	[66] 2012
NC, AD, both genders (68–90 years) *n* = 50 (Australia)	-Balance -Risk of falls -Gait	↑ Risk of falls. ↑ Alterations in dynamic and static balance. ↑ Gait disturbances such as turning and dual tasks.	[82] 2012
**Animal model studies**			
Transgenic *3xTg*-AD mice, females; 2, 4, 18, and 20 months (*n* = 36)	-NP signs -Muscle mass -NMJ -Mitochondrial complexes at the muscle level	↑ Aβ and tau in the brain, spinal cord, nerve, and muscle in early and late stages. ↑ Muscle atrophy in the early stages exacerbated in the advanced stages. -No changes in contractile proteins or motor neurons. ↑ Denervation in the NMJ in late stages. ↑ ROS at the muscle level and alterations in mitochondrial respiratory complexes. -Activation of the TGF-β pathway related to atrophy.	[19] 2022
Transgenic *3xTg*-AD mice, males; 6, 12, and 16 months (*n* = 45)	-Posture -Coordination -Gait -Balance	↓ Stride length, speed, and cadence from an early age. -Speed and cadence correlate with postural alterations. ↑ Motor pathway alterations progress with age. ↑ Alterations in posture and coordination in advanced stages of the disease.	[93] 2022
Transgenic *3xTg*-AD mice, males, 6 months (*n* = 30)	-Balance -Coordination -Sensorimotor activity	↑ Balance disturbance. ↑ Coordination problems. ↓ Sensorimotor activity from the early stages.	[94] 2022
Transgenic *APP Tg2576* mice, females, 6 months (*n* = 10)	-NMJ - Cholinergic activity	↓ Neuronal innervation and synaptic area. ↓ Chat. ↓ Cholinergic innervation at the muscle level.	[95] 2021
Transgenic *3xTg*-AD mice, males; 6, 12 and 16 months (*n* = 45)	-Gait -Strength -Posture -Hypertrophy -Locomotor activity -Posture	↓ Muscle strength in the intermediate and late stages of the disease. -Changes in motor performance from the early stages of the disease. ↓ Gait speed, cadence, and step length that progress with the stages of the disease. ↑ Atrophy more evident in the late stages of the disease. ↑ Presence of postural changes (hyperkyphosis) in advanced stages.	[53] 2021
Transgenic *5xFAD* mice, females, and males, 3–16 months	-Locomotor activity -Balance -Strength -Coordination	↓ Locomotor activity, coordination, strength, and balance from the intermediate stages (9 months) and worsen in the late stages (16 months). -No differences between sexes.	[96] 2020
Transgenic *TgCRND8* mice; 5, 7, 10, and 18 months (*n* = 24)	-NP signs -Axonopathy	↑ Dilated corticospinal axons at 7 months and age-dependent. ↑ βA in the spinal cord at 10 months. ↑ Axonal dystrophies and dense vesicles.	[97] 2019
Transgenic *Tg4-42* mice, both genders, 3 and 7 months (*n* = 90)	-Coordination -Balance - Cerebellar metabolic activity	↑ Balance and motor coordination problems in aged mice. ↓ Locomotor activity. ↓ Cerebellar metabolism PET/MRI with 18F-FDG.	[98] 2019
Transgenic *APP/PS* mice, both genders; 3, 6, 9, 12, and 18 months (*n* = 60)	-Muscle mass -Strength -Myostatin	-Correlation of muscle atrophy and memory impairment. ↑ Atrophy at 12 months but significant from 9 months. ↑ Myostatin in gastrocnemius at 12 months. -Removal of myostatin increased grip strength and muscle mass.	[99] 2019
Transgenic *APP/PS1* mice, 7–8 months (*n* = 43)	-NMJ	↑ Synaptic alterations at the muscular level. ↓ Quantum content and amplitude of terminal plate potentials. ↑ Synaptic vesicle recycling time. - Disordered neurosecretion and recycling of synaptic vesicles at presynaptic nerve endings.	[100] 2018
Transgenic *McGill-R-Thy1-APP* transgenic rats, males, 4–7 months (*n* = 20)	-Locomotor activity -Coordination -Balance	-No changes in locomotor activity. ↑ Alterations in coordination and static-dynamic balance.	[101] 2018
Transgenic *TgCRND8* mice, both genders, 2 months (*n* = 23)	-Balance -Gait -Synaptic plasticity in the cerebellum	↑ Motor coordination and balance deficits. ↓ Step length. ↑ Altered noradrenergic modulation at the parallel synapse between fiber and Purkinje cells. ↑ Dysfunction of cerebellar circuits.	[102] 2018
Transgenic *5xFAD*, mice, both genders; 11, 19, and 27 weeks (*n* = 34)	-NP signs -Axonopathy and myelopathy	↑ βA in the spinal cord from 11 weeks and age-dependent. ↑ βA in the gray and white matter of the mouse spinal cord. -No changes in motor neurons. ↑ Myelinopathy in the spinal cord in old age.	[103] 2017
Transgenic *3xTg*-AD mice, males; 3, 6, and 12 months (*n* = 18)	-Muscle -Mitochondrial alterations -Cholinergic system	↑ Alterations in acetylcholinesterase–catalase activity from 3 months of age. ↑ βA muscle at 6 months and more pronounced at 12. ↑ Alterations of mitochondrial respiratory complexes at 6 months.	[104] 2015

Abbreviations: *3xTg*-AD: Triple-transgenic Alzheimer’s disease model, Aβ42: Beta-amyloid peptide 42, AD: Alzheimer’s disease, APP: Amyloid precursor protein, *APP/PS*: Amyloid precursor protein/presenilin, *APP/PS1*: Amyloid precursor protein/presenilin 1, APOE ε4: Apolipoprotein E ε4 allele, Chat: Choline acetyltransferase, CRF: Cardiorespiratory fitness, FDG: Fluorodeoxyglucose, LBD: Lewy body dementia, MCI: Mild cognitive impairment, *McGill-R-Thy1-APP:* Transgenic rat model overexpressing human APP, MRI: Magnetic resonance imaging, NMJ: Neuromuscular junction, NC: Normal cognition, NP signs: Neuropsychiatric signs, PET: Positron emission tomography, ROS: Reactive oxygen species, TGF-β: Transforming growth factor beta, *Tg2576*: Transgenic model overexpressing APP, *Tg4-42*: Transgenic model with APP mutations, *TgCRND8*: Transgenic model with APP mutations, VD: Vascular dementia, *5xFAD*: Transgenic model with five familial AD mutations, ↑: Increase, and ↓: Decrease.

## Data Availability

No new data were created or analyzed in this study.
